# Supplementary figures and images for: Multiparametric Optimization of Human Primary B‐Cell Cultures Using Design of Experiments
Source: Scand J Immunol. 2025 Jul 28;102(2):e70043. doi: 10.1111/sji.70043 (PMC12304294; doi:10.1111/sji.70043)

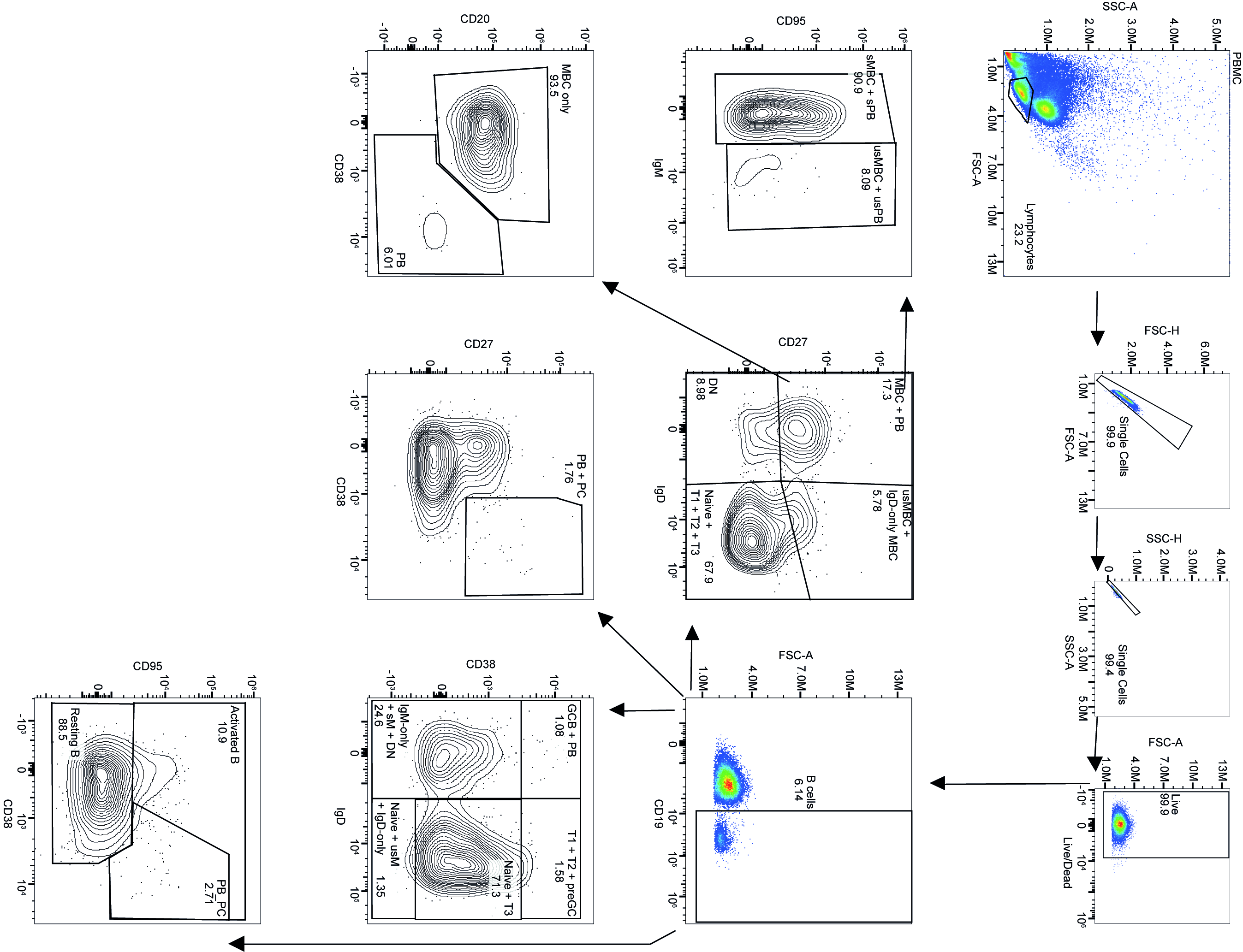

Supplement: Supplementary file 1 — Figure S1. Flow cytometry gating strategy. Stepwise gating of human PBMCs, exemplifying the lymphocyte/singlet‐FSC/singlet‐SSC/live/CD19+ hierarchy used in subsequent analyses. [file SJI-102-e70043-s005.tif]

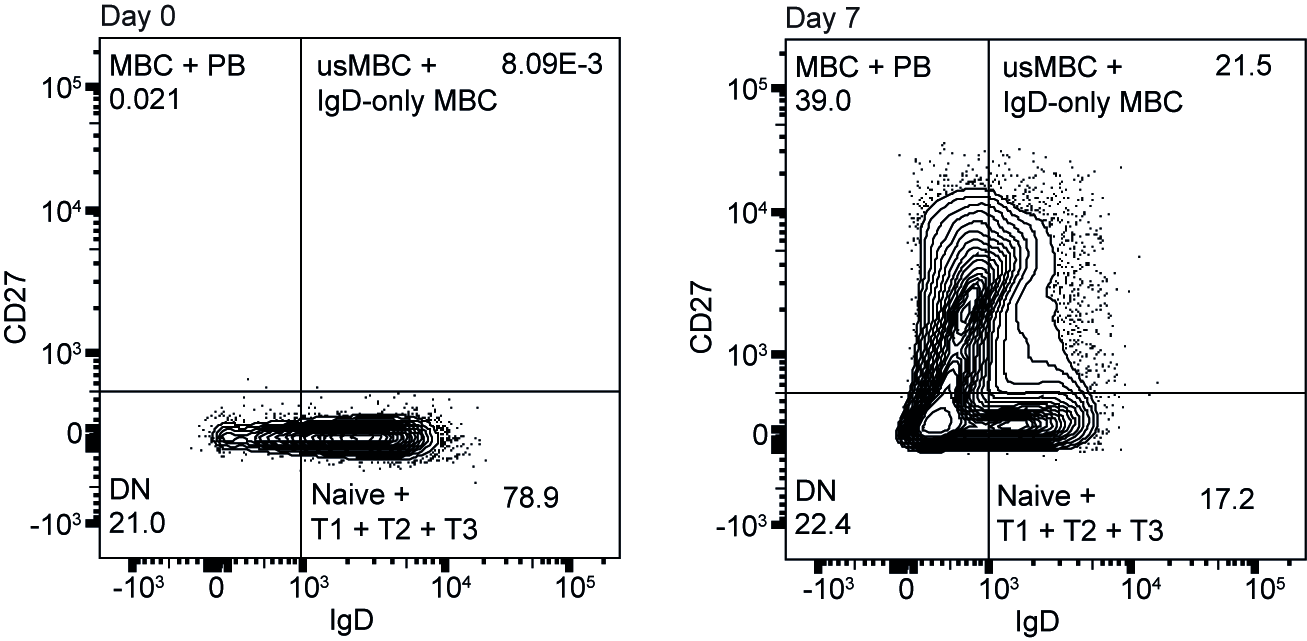

Supplement: Supplementary file 2 — Figure S2. Naïve B‐cell activation with NHDF03 feeder cells. Flow cytometric comparison of naïve B cells at Day 0 versus 7 when co‐cultured with NHDF03‐hTert‐CD40L cells, illustrating progressive upregulation of B‐cell activation markers. [file SJI-102-e70043-s004.tif]

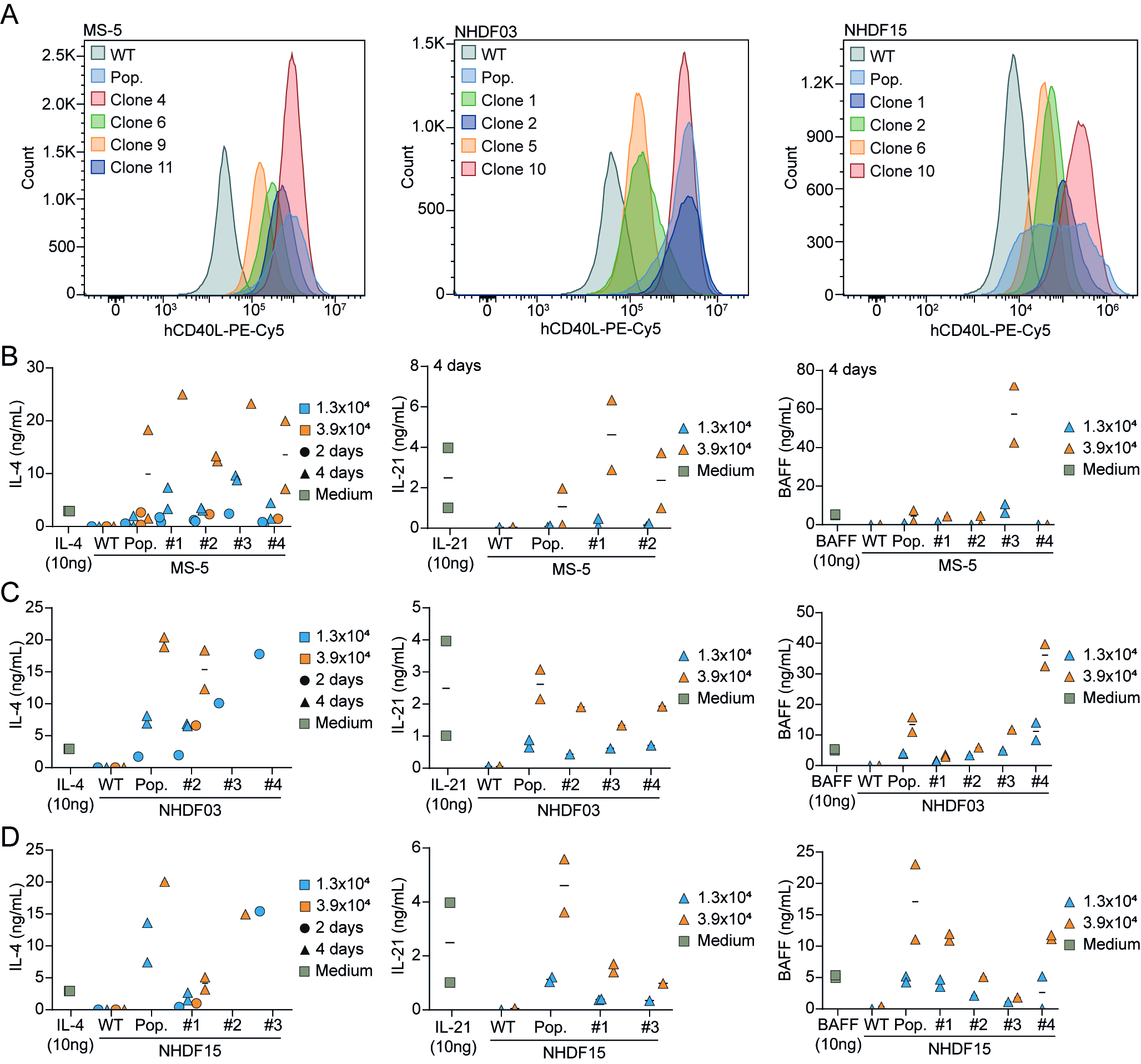

Supplement: Supplementary file 3 — Figure S3. Generation of feeder cells from murine MS‐5 and human NHDF03/NHDF15 lines. (A) CD40L display of MS‐5, NHDF03 and NHDF15 measured by flow cytometry. (B‐D) ELISA confirmation of IL‐4, IL‐21 and BAFF secretion by transgenic MS‐5 (B), NHDF03 (C) and NHDF15 (D) feeder cells. [file SJI-102-e70043-s008.tif]

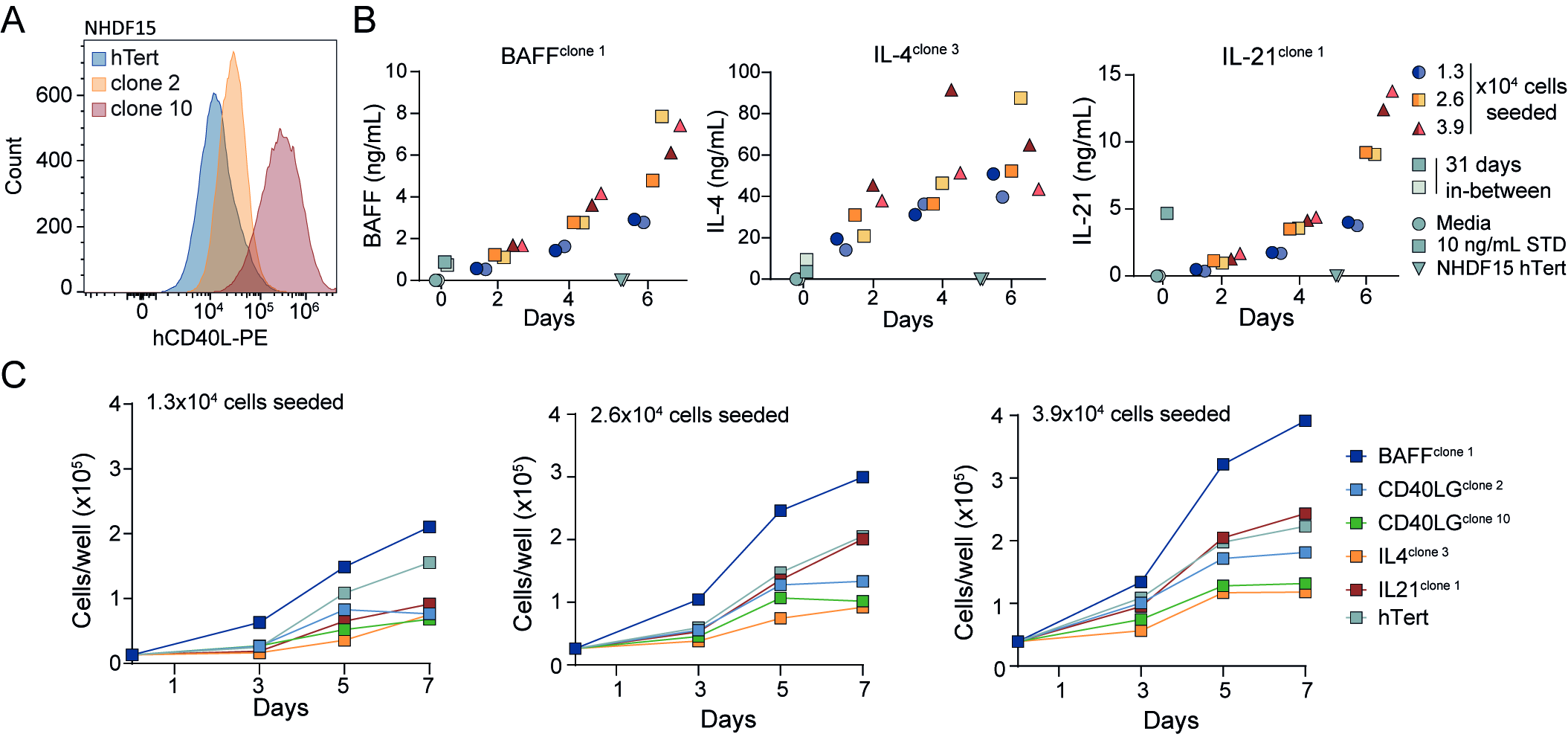

Supplement: Supplementary file 4 — Figure S4. Characterisation of NHDF15 transgenic clones. (A) CD40L expression on NHDF15 clones 2 (N.40‐low) and 10 (N.40‐high), as well as hTert negative controls, by flow cytometry. (B) ELISA‐based measurement of IL‐4, IL‐21 and BAFF secretion over time. (C) Longitudinal assessment of feeder‐cell proliferation by flow cytometry. [file SJI-102-e70043-s006.tif]

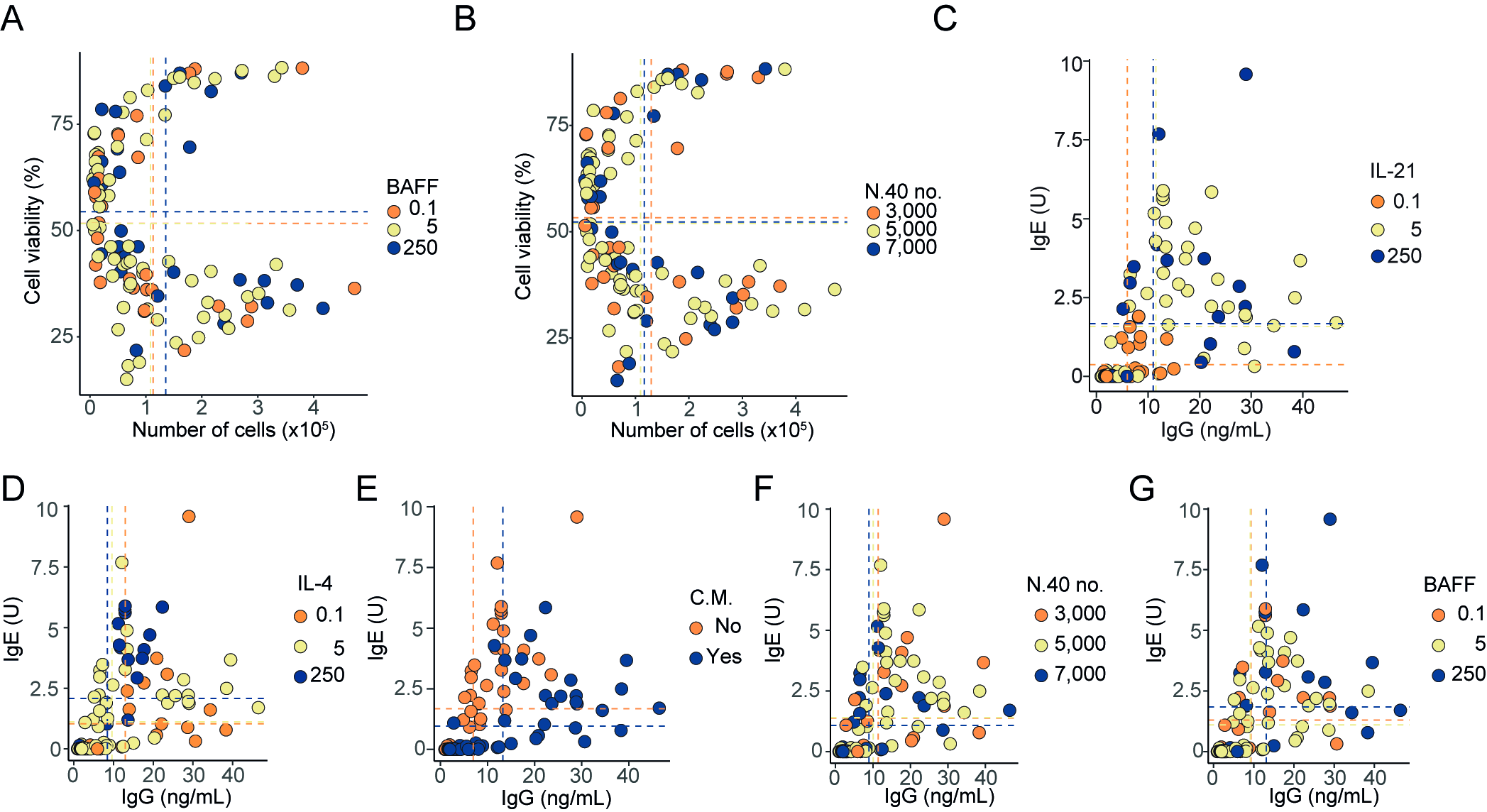

Supplement: Supplementary file 5 — Figure S5. DOE 3 supplementary findings. (A–C) Proliferation and viability of each sample from DOE 3 where colour and mean indicated by dotted lines are specific for BAFF (A), number of N.40 cells (B), and IL‐21 (C). (D–G) TRIFMA‐measured IgG and IgE in culture medium, colour‐coded for the level of IL‐4 (D), change of medium (E), number of N.40 cells (F) and level of BAFF (G). C.M. indicates medium change. [file SJI-102-e70043-s001.tif]

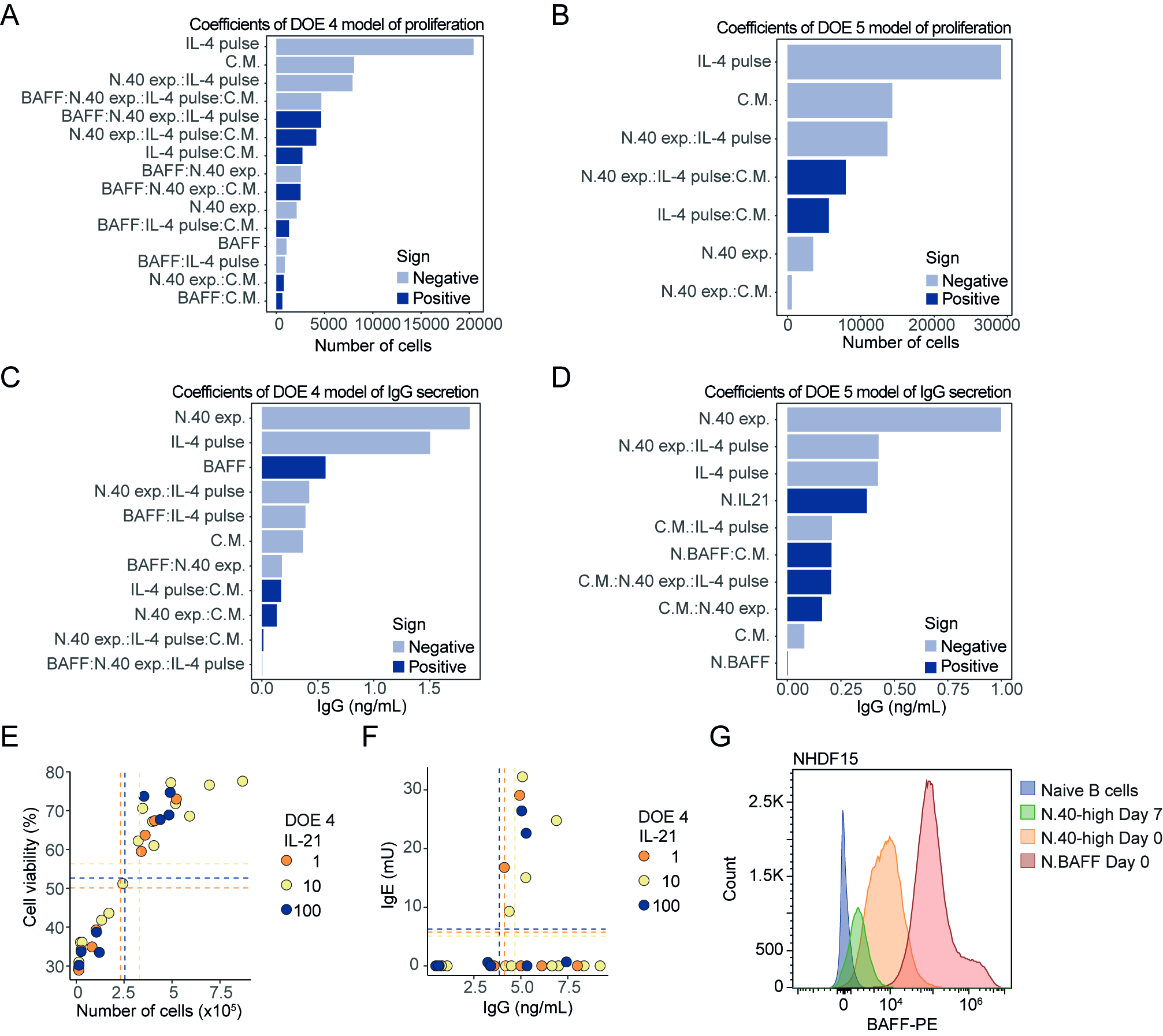

Supplement: Supplementary file 6 — Figure S6. Supplementary findings for DOE 4 and 5 comparison of cytokine delivery method. (A, B) Pareto plot of parameter effect sizes on B‐cell proliferation in DOE 4 (A) and DOE 5 (B). (C, D) Pareto plot of parameter effect sizes on IgG detection in the medium in DOE 4 (C) and 5 (D), measured by TRIFMA. (E) Raw data plotting of B‐cell number and viability after 8 days of iGB culturing, with dotted lines showing the mean of the respective factor. Colour‐coding indicates the number of seeded IL‐21‐expressing feeder cells in DOE 4. (F) As (E) but for measured IgG and IgE. (G) Flow cytometric quantification of membrane‐bound BAFF on N.40‐high NHDF clones either taken from 7‐days of iGB co‐culturing or uncultured. Naïve B cells were included as a negative control for membrane‐bound BAFF and BAFF‐expressing NHDFs, N.BAFF, as positive control. [file SJI-102-e70043-s003.tif]

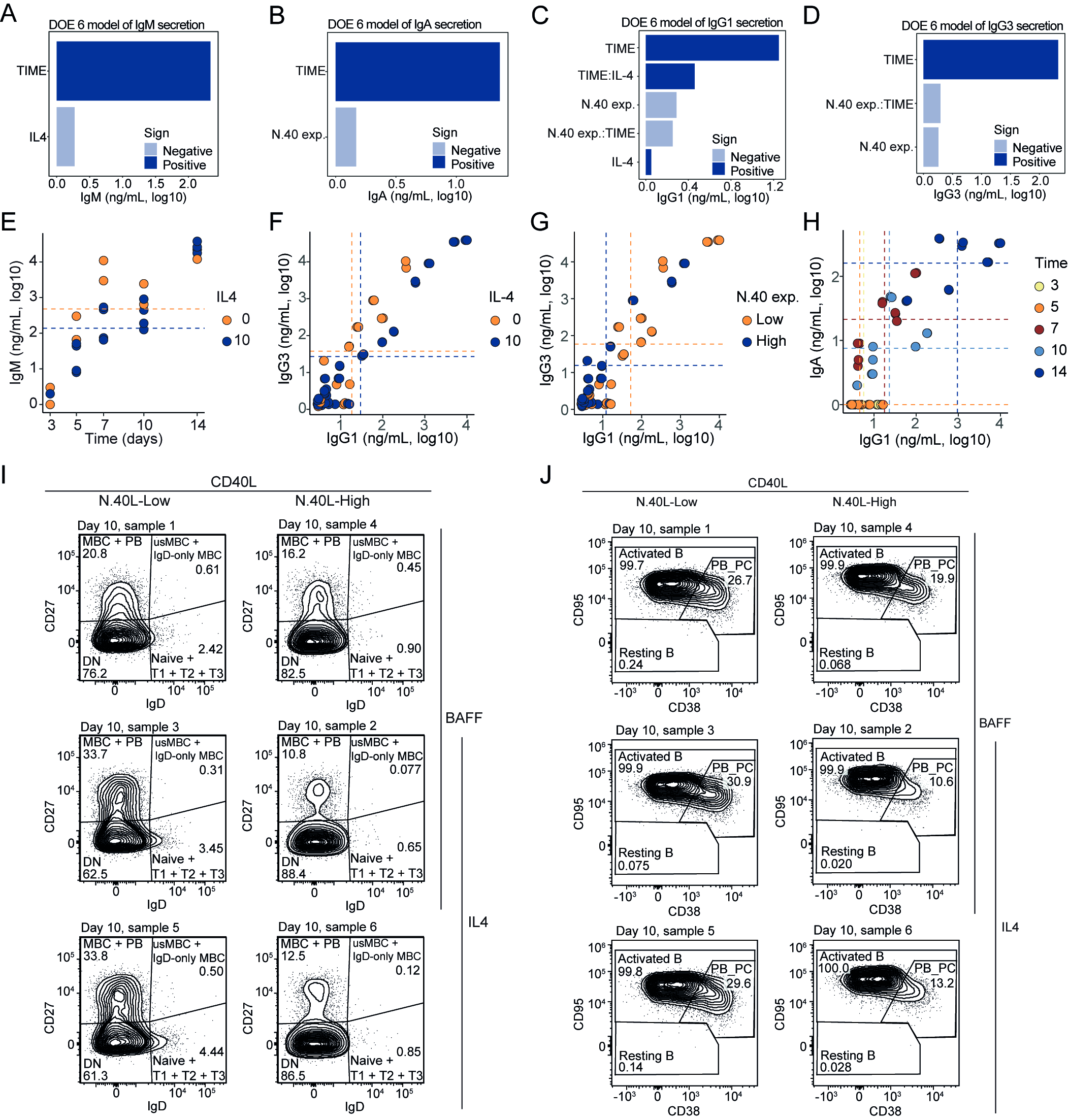

Supplement: Supplementary file 7 — Figure S7. DOE 6 supplementary findings. (A–D) Pareto plot of parameter effect sizes on IgM (A), IgA (B), IgG1 (C) and IgG3 (D) detection in the medium of DOE 6 cultures measured by TRIFMA. (E) Measured IgM over time in culture medium of each sample from DOE 6 where colour and mean, indicated by dotted lines, are specific for the level of IL‐4. (F) as (E) but for measuring IgG1 and IgG3. (G) as (F) but dotted lines indicate N.40 expression. (H) as (E–G) but for measured IgG1 and IgA, with dotted lines specific for time. (I) Flow plotting IgD versus CD27 of CD19+ live singlet lymphocytes after 10 days of naïve B‐cell iGB culturing to illustrate the difference of their activation and differentiation depending on the stimuli provided, with the feeder‐cell expression level of CD40L being a very apparent contributor. (J) as (I) but plotting CD38 versus CD95. [file SJI-102-e70043-s002.tif]
