# Supplementary material for: Multiparametric Optimization of Human Primary B‐Cell Cultures Using Design of Experiments
Source: Scand J Immunol. 2025 Jul 28;102(2):e70043. doi: 10.1111/sji.70043 (PMC12304294; doi:10.1111/sji.70043)
Supplement: Supplementary file 8 — Appendix S1. [file SJI-102-e70043-s007.docx]

## **Supplementary Table 1 – Primers used for Gibson assembly**

| **Primer** | **Sequence (5’ -> 3’)** |
| --- | --- |
| pCCL-PGK-hTert-IRES-Hygro | |
| hTERT fragment, forward | GGCCTTTCGACCTCTAGCGGTGTCGTGAGGATCCATGC |
| hTERT fragment, reverse | AGGGAAACCGTTGCTAGC |
| Hygro fragment, forward | AAGCTAGCAACGGTTTCCCT |
| Hygro fragment, reverse | CCAGAGGTTGATTATCGGAATTCCCGCCCGGGCTATTCCTTTGCCCTC |
| pCCL-PGK-IL4-IRES-Puro | |
| IL4-IRES-Puro fragment, forward | CTCCGGGCCTTTCGACCTCTAGCGGTCTAGAAGCGCTGGATCC |
| IL4-IRES-Puro fragment, reverse | CCAGAGGTTGATTATCGGAATTCCCTCTCGAGATTAATCAGGCACCGGGC |
| pLV-PGK-mCherry-BHGpA_CMV-TNFSF13B-WHV | |
| BAFF fragment 1^st^, forward | AGTGAACCGTCAGATCTTTTCTAGACACCATGGATGACTCCAC |
| BAFF fragment 1^st^, reverse | GGTTGATTATCGGAATTCCCTCGAGTCACAGCAGTTTCAATGC |
| pCCL-PGK-BAFF-IRES-Puro | |
| BAFF fragment 2^nd^, forward | TGTCGACGATATCTTCGAAGGACACCATGGATGACTCC |
| BAFF fragment 2^nd^, reverse | ACGGCCGCTATGCTTTACTGGGATCCTCACAGCAGTTTCAATGC |
| pCCL-PGK-IL21-IRES-Puro | |
| IL21 fragment, forward | TGTCGACGATATCTTCGAAGACGCGTGCCACCATGAGATC |
| IL21 fragment, reverse | ACGGCCGCTATGCTTTACTGTTAATTAACTAAGAGTCCTCTGACCCATG |

## **Supplementary Table 2 - Flow Cytometry Antibodies**

| **Anti-human antibody** | **Clone, Company, Cat#** |
| --- | --- |
| Purity QC (used following every MACS purification) | |
| Mouse α-CD19-PE | Clone HIB19, BD, 555413 |
| Mouse α-CD20-BV421 | Clone 2H7, BD, 562873 |
| Mouse α-IgD-PE/Cy7 | Clone IA6-2, BD, 561314 |
| Mouse α-CD8-PerCP/Cy5.5 | Clone SK1, BD, 565310 |
| Mouse α-CD4-AF647 | Clone RPA-T4, BD, 557707 |
| Mouse α-CD14-BV605 | Clone M5E2, BD, 564055 |
| Mouse α-CD16-FITC | Clone 3G8, BD, 555406 |
| Viability Dye eFluor 780 | eBioscience, 65-0865-14 |
| Human BD Fc Block | Clone Fc1, BD, 564220 |
| B indentity analysis (Seen in Fig 1, 6, S1, S2, S7) | |
| Mouse α-CD19-FITC | Clone HIB19, BD, 555412 |
| Mouse α-CD20-BV421 | Clone 2H7, BD, 562873 |
| Mouse α-CD24-PE-CF594 | Clone ML5, BD, 562405 |
| Mouse α-CD27-BV786 | Clone L128, BD, 563327 |
| Mouse α-CD38-APC | Clone HIT2, BD, 555462 |
| Mouse α-CD95-PE | Clone DX2, BD, 561976 |
| Mouse α-IgD-PE-Cy7 | Clone IA6-2, BD, 561314 |
| Mouse α-IgM-BV605 | Clone G20-127, BD, 562977 |
| Viability Dye eFluor 780 | eBioscience, 65-0865-14 |
| Human BD Fc Block | Clone Fc1, BD, 564220 |
| Viability, Proliferation (Seen in Fig 1-5, S5, S6) | |
| Propidium Iodide | Invitrogen, BMS500PI |
| CD40L quantification (Seen in Fig S3 and S4, respectively) | |
| Mouse α -CD154 (CD40L)-PE/Cy5 | Clone 24-31, Invitrogen, 15-1548-42 |
| Mouse α -CD154 (CD40L)-PE | Clone TRAP1, BD, 555700 |
| Membrane-bound BAFF quantification (Seen in Fig S6) | |
| Mouse α -CD154 (CD40L)-BV421 | Clone TRAP1, BD, 563886 |
| Mouse α-CD19-FITC | Clone HIB19, BD, 555412 |
| Mouse α -CD257 (BAFF)-PE | Clone 1D6, BioLegend, 366505 |
| Mouse α-CD38-APC | Clone HIT2, BD, 555462 |
| Mouse α-CD20-AF700 | Clone 2H7, BD, 560631 |
| Viability Dye eFluor 780 | eBioscience, 65-0865-14 |
| Human BD Fc Block | Clone Fc1, BD, 564220 |
